# Anti-inflammatory microRNA-146a protects mice from diet-induced metabolic disease

**DOI:** 10.1371/journal.pgen.1007970

**Published:** 2019-02-15

**Authors:** Marah C. Runtsch, Morgan C. Nelson, Soh-Hyun Lee, Warren Voth, Margaret Alexander, Ruozhen Hu, Jared Wallace, Charisse Petersen, Vanja Panic, Claudio J. Villanueva, Kimberley J. Evason, Kaylyn M. Bauer, Timothy Mosbruger, Sihem Boudina, Mary Bronner, June L. Round, Micah J. Drummond, Ryan M. O’Connell

**Affiliations:** 1 Department of Pathology, University of Utah, Salt Lake City, Utah, United States of America; 2 Department of Biochemistry, University of Utah, Salt Lake City, Utah, United States of America; 3 Bioinformatics, Huntsman Cancer Institute and University of Utah, Salt Lake City, Utah, United States of America; 4 Department of Nutrition and Integrative Physiology, University of Utah, Salt Lake City, Utah, United States of America; 5 Department of Physical Therapy and Athletic Training, University of Utah, Salt Lake City, Utah, United States of America; Mount sinai school of medicine, UNITED STATES

## Abstract

Identifying regulatory mechanisms that influence inflammation in metabolic tissues is critical for developing novel metabolic disease treatments. Here, we investigated the role of microRNA-146a (miR-146a) during diet-induced obesity in mice. miR-146a is reduced in obese and type 2 diabetic patients and our results reveal that miR-146a-/- mice fed a high-fat diet (HFD) have exaggerated weight gain, increased adiposity, hepatosteatosis, and dysregulated blood glucose levels compared to wild-type controls. Pro-inflammatory genes and NF-κB activation increase in miR-146a-/- mice, indicating a role for this miRNA in regulating inflammatory pathways. RNA-sequencing of adipose tissue macrophages demonstrated a role for miR-146a in regulating both inflammation and cellular metabolism, including the mTOR pathway, during obesity. Further, we demonstrate that miR-146a regulates inflammation, cellular respiration and glycolysis in macrophages through a mechanism involving its direct target *Traf6*. Finally, we found that administration of rapamycin, an inhibitor of mTOR, was able to rescue the obesity phenotype in miR-146a-/- mice. Altogether, our study provides evidence that miR-146a represses inflammation and diet-induced obesity and regulates metabolic processes at the cellular and organismal levels, demonstrating how the combination of diet and miRNA genetics influences obesity and diabetic phenotypes.

## Author summary

Obesity and metabolic disease are on the rise throughout the world, creating a need for research into the interaction between diet and genetics. It is known that chronic inflammation contributes to obesity, but it is not well understood how inflammation and inflammatory genes are controlled to prevent obesity. Here we found that a specific microRNA, miR-146a, controls inflammation and prevents obesity onset when mice are fed a high-fat diet. In the absence of miR-146a, mice become obese and develop diabetic symptoms in a diet-dependent manner. We found that miR-146a is highly expressed in immune cells and regulates the inflammatory function of macrophages within fat tissue. Within macrophages, we identified metabolic pathways regulated by miR-146a that could contribute to its protective role during DIO, and show that blockade of mTOR using rapamycin could reverse the obesity phenotype in miR-146a-/- mice. Human studies have previously shown that miR-146a expression is decreased in obese and diabetic patients, and our work suggests that miR-146a is mechanistically involved in the development of obesity and therefore can be a potential therapeutic target. Taken together, our work demonstrates that miRNA genetics and lifestyle are both important, contributing factors to diet-induced metabolic disease.

## Introduction

Obesity incidence has reached epidemic rates in recent years, now affecting over one-third of the adult population in the United States and rising in incidence around the world [1]. Obesity and its comorbidities, including diabetes, heart disease, stroke, cancer, and infections, account for the second leading cause of preventable death in the United States, placing a large burden on the healthcare system and diminishing the health and life expectancy of affected individuals [2, 3]. Chronic, low-grade inflammation and metabolic dysregulation are at the center of obesity pathogenesis and progression, but the mechanisms underlying this dysregulation are not fully understood. Insights into how inflammation and metabolism contribute to obesity could aid in developing therapies and combating further spread of this condition.

Among regulators of metabolic pathways and inflammation are microRNAs (miRNAs). miRNAs are ~22 nucleotide noncoding RNAs that post-transcriptionally regulate mRNA targets, thus inhibiting protein expression. Roles for miRNAs in inflammation and immunity have been widely studied, and many particular miRNAs are essential for proper immune system function [4–6]. One such miRNA, miR-146a, is induced during inflammatory responses and acts to dampen inflammation in multiple contexts, including cancer [7–9], autoimmunity [10, 11], immunization [12], and intestinal homeostasis [13]. Importantly, miR-146a has been shown to function in human adipose tissue during inflammation [14], and its expression is reduced in obese and type 2 diabetic (T2D) patients [15–19], suggesting a role for miR-146a in obesity.

Here, we show that genetic deletion of miR-146a in mice results in obesity, fatty liver disease, and dysregulated glucose levels during diet-induced obesity (DIO). These mice exhibit elevated inflammation in metabolic tissues both before and during metabolic disease. We found that miR-146a is expressed within adipose tissue and regulates the accumulation of inflammatory foci that resemble crown-like structures during a high-fat diet (HFD). Further, miR-146a is highly expressed in the stromal vascular fraction (SVF) of the adipose tissue, suggesting a role for miR-146a within immune cells within this compartment. RNA-sequencing analysis revealed that miR-146a regulates not only inflammatory pathways, but also metabolic pathways including mTOR in adipose tissue macrophages (ATMs), a role not previously identified. Further assessment found that miR-146a regulates both the inflammatory response and metabolic state of macrophages through a mechanism involving repression of its target, *Traf6*. Finally, we found that inhibition of mTOR using rapamycin was able to reverse the obesity phenotype in miR-146a-/- mice. Altogether, these data indicate that miR-146a functions to limit diet-induced metabolic disease in mice.

## Results

### miR-146a limits obesity during a high fat diet

**Fig 1:**
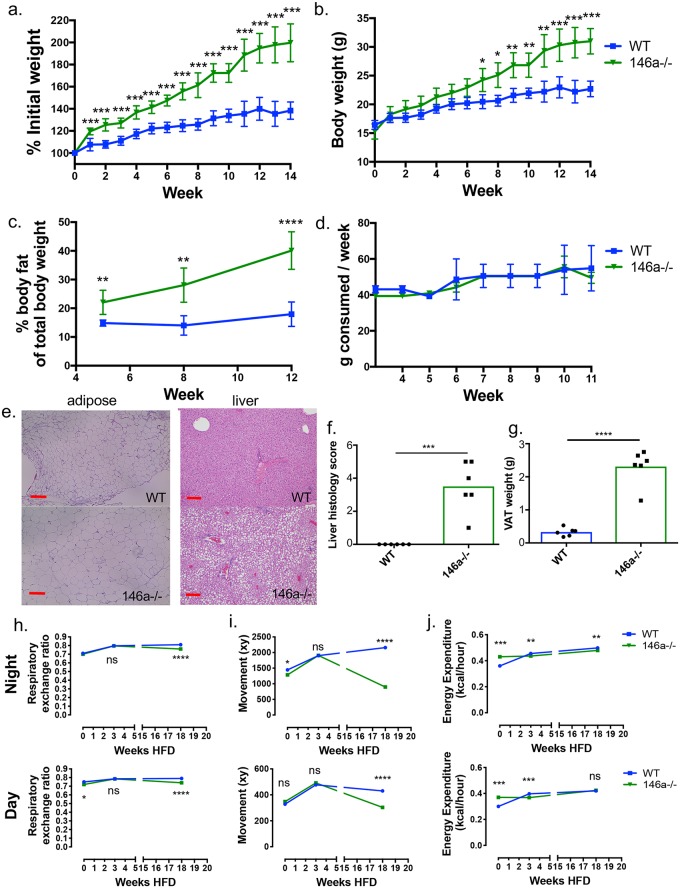
miR-146a protects against weight and fat gain and liver steatosis during diet-induced obesity. (A) C57BL/6 WT and miR-146a-/- mice were placed on HFD. Measure of percent weight over time of WT (blue) and miR-146a-/- mice (green), based on initial weight. (B) Body weight (g) of WT and miR-146a-/- mice over time. (C) Percent body fat of WT and miR-146a-/- at time points 5, 8, and 12 weeks HFD. (D) Amount of chow consumed per mouse each week over time of diet. (E) H&E staining of representative sections of VAT (left) and liver (right) at week 14 of diet treatment. miR-146a-/- VAT is hypertrophied and liver has severe steatosis compared to WT. 10x magnification; red line indicates 200 μm. (F) Histology scores of H&E stained liver tissues, measuring steatosis and inflammatory foci within livers at 14 weeks HFD. (G) Weight of VAT at 14 weeks HFD. (H) Average respiratory exchange ratio of mice on HFD for 0, 3, or 18 weeks, calculated by V_O2_ and V_CO2_ max levels, measured at day and night. (I) Average XY movement of mice, measured at day and night. (J) Average energy expenditure by mice in kcal/hour, measured at day and night. Data are shown as mean ± SEM (n = 6), 5 repeats were performed for the HFD experiments assessing weight gain and adiposity. p-value was calculated using two-tailed Student’s t-test. *p<0.05; **p<0.01; ***p<0.001; ****p<0.0001. See also S1 Fig.

In order to study the role of miR-146a in obesity and metabolic disease, we fed miR-146a-/- and C57BL/6 WT mice HFD. Over time, miR-146a-/- mice gained significantly more body weight than WT controls as a measure of percent initial weight (Fig 1a, S1a Fig) as well as weight in grams (Fig 1b). NMR body composition analysis revealed that miR-146a-/- mice gained significantly more body fat than WT mice, with a body composition of ~40% body fat by week 12 of the HFD compared to ~20% fat for WT (Fig 1c). We observed no significant difference in weight gain or fat accumulation between WT and miR-146a-/- mice fed a normal chow diet (NCD) (S1b–S1d Fig). Further indicative of obesity and metabolic dysregulation, miR-146a-/- mice on HFD developed elevated serum Leptin protein levels (S1e Fig). Increased weight gain was seen in both male and female miR-146a-/- mice (S1f Fig). To account for possible colony-specific microbiota differences and as further confirmation, miR-146a-/- mice from Jackson Labs also showed increased weight gain compared to WT Jackson C57BL/6 controls (S1g and S1h Fig). The significant weight and fat gain by miR-146a-/- HFD mice was not caused by an increase in food consumption, as the groups had similar food intake over the course of the diet (Fig 1d and S1i Fig).

Upon histological examination of the adipose tissue, miR-146a-/- mice fed HFD displayed varying degrees of hypertrophied adipocytes compared to WT mice [20] (Fig 1e, left). Liver histological analysis showed steatosis in miR-146a-/- HFD mice that was not apparent in WT mice (Fig 1e, right). Further, liver histology scores, which measure portal inflammation and steatosis as previously outlined [21], revealed that miR-146a-/- HFD mice had significantly increased liver histology scores compared with WT controls (Fig 1f), indicating miR-146a-/- HFD mice have worsened metabolic disease in the liver. Visceral white adipose tissue (VAT) pads from miR-146a-/- mice on HFD weighed significantly more than WT VAT pads (Fig 1g).

Mice were placed in metabolic chambers at 0, 3, or 18 weeks during HFD to measure phenotypic parameters before and during onset of obesity. At 18 weeks HFD, miR-146a-/- mice showed a decreased respiratory exchange ratio compared with WT mice, while minimal differences were observed at earlier time points (Fig 1h), indicating that miR-146a-/- mice began using more fat for energy later in the time course. No difference in movement was observed at 0 or 3 weeks HFD, but by 18 weeks HFD miR-146a-/- mice displayed significantly decreased movement compared to WT controls likely a result of their increased adiposity and weight gain (Fig 1i). Although subtle, altered energy expenditure further suggested metabolic dysregulation (Fig 1j).

To determine whether brown adipose tissue (BAT) played a role, we removed BAT from young, untreated animals and determined gene expression for BAT activation genes (S2a Fig), lipogenesis genes (S2b Fig), and inflammatory immune genes (S2c Fig). We did not find significant differences between WT and miR-146a-/- BAT tissue in terms of gene expression, while the removed BAT tissue weighed the same between the two groups (S2d Fig). We also determined that miR-146a was expressed in BAT tissue from WT mice and absent in BAT from miR-146a-/- mice (S2e Fig). Following HFD, we again assessed expression of relevant genes and saw no significant differences between WT and miR-146a-/- (S2f Fig). These data suggest that mouse BAT function is not playing a major role in the phenotype. Taken together, these data indicate that the combination of a miR-146a deficiency and HFD work together to exacerbate weight gain and metabolic disease in this model of DIO.

### miR-146a protects mice from hyperglycemia during HFD

**Fig 2:**
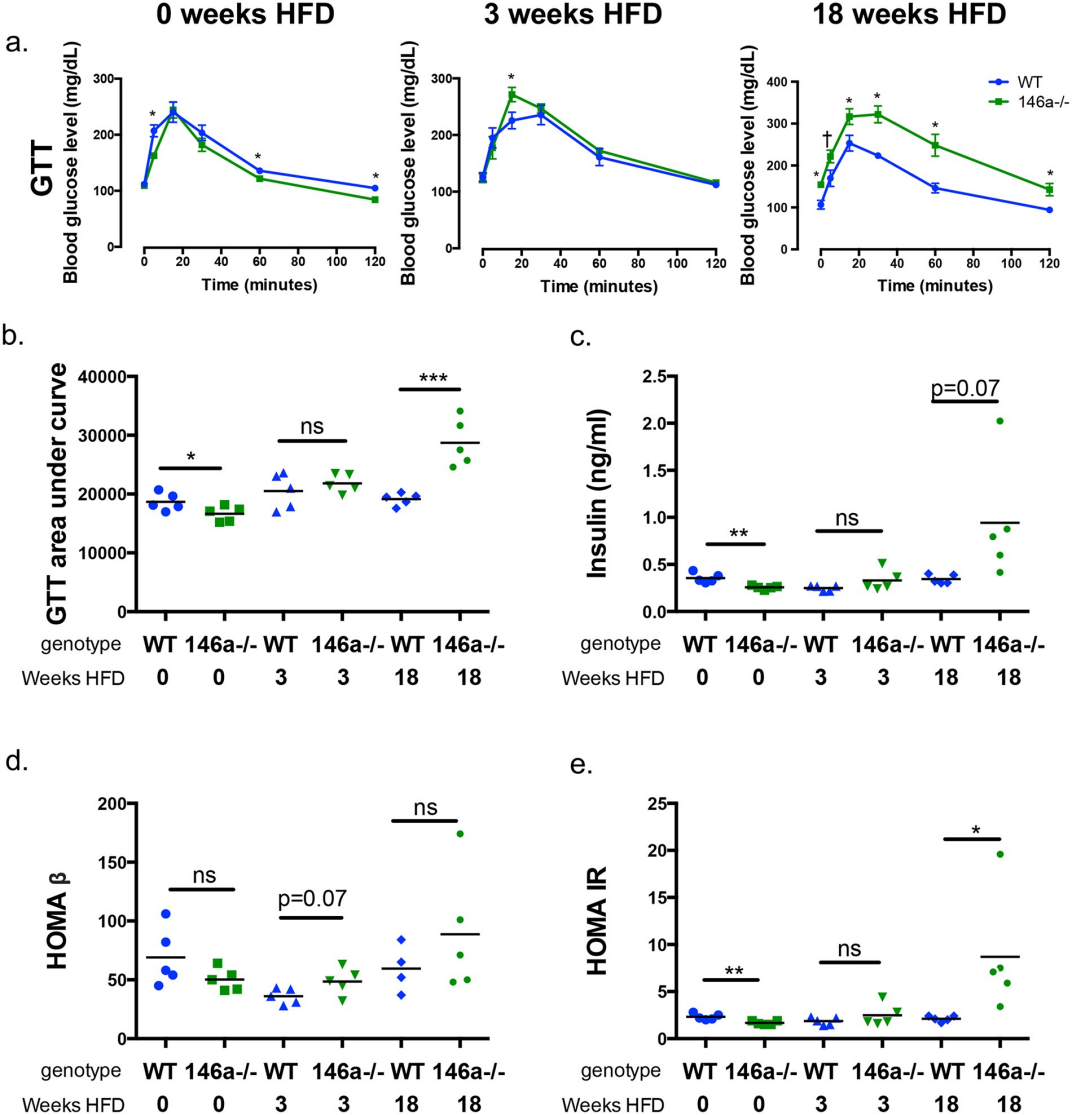
miR-146a is required for protection from a diabetic phenotype during diet-induced obesity. All measurements were performed in WT (blue) and miR-146a-/- (green) mice at 0, 3, and 18 weeks HFD. (A) GTTs measuring blood glucose levels, following injection of glucose at 0 minutes and measured over time for 120 minutes. (B) Area under curve of GTTs. (C) Serum insulin levels, measured via ELISA. (D) HOMA-β levels, measuring beta cell function. (E) HOMA-IR levels, measuring insulin resistance. Data are shown as mean ± SEM (a) or as individual mice (b-e) (n = 5). p-value was calculated using two-tailed Student’s t-test. *p<0.05; **p<0.01; ***p<0.001; ns = not significant. See also S3 Fig.

Obesity is closely associated with glucose dysregulation, and recent reports found decreased miR-146a levels in PBMCs and serum of patients with T2D [15, 16, 18]. Thus, we examined glucose homeostasis in miR-146a-/- mice during DIO. miR-146a-/- mice fed HFD displayed higher blood glucose during glucose tolerance tests (GTTs) than WT controls, or than either group on NCD (S3a and S3b Fig). GTTs were performed on fasted WT and miR-146a-/- mice at 0, 3, or 18 weeks HFD to determine if glucose phenotypes precede or succeed obesity in miR-146a-/- mice. Results indicate little difference between WT and miR-146a-/- mice in blood glucose at 0 and 3 weeks of HFD (Fig 2a and 2b). However, after 18 weeks, miR-146a-/- mice had significantly higher blood glucose levels than WT mice at resting and during GTT, with an increase in the GTT area under the curve (Fig 2a and 2b). These data indicate that miR-146a-/- mice are glucose tolerant when on NCD and during early stages of DIO but lose the ability to properly regulate glucose after adiposity has begun.

miR-146a-/- and WT mice had comparable fasting serum insulin levels at 0 and 3 weeks HFD, yet miR-146a-/- mice had notably higher levels of serum insulin at 18 weeks HFD (Fig 2c), revealing that miR-146a-/- mice still make insulin. Increased insulin levels can indicate insulin resistance, as hyperinsulinemia is a common symptom in T2D patients [22]. HOMA-β and HOMA-IR, which are based on fasting blood glucose and insulin levels and measure pancreatic beta cell function and insulin resistance, respectively, were calculated. Consistent with our observation that miR-146a-/- mice can still produce insulin, we saw no difference in HOMA-β function between WT and miR-146a-/- mice (Fig 2d); additionally, pancreatic architecture of HFD-fed WT and miR-146a-/- mice appeared normal upon H&E staining (S3c Fig). On the other hand, HOMA-IR was increased in the miR-146a-/- HFD group at 18 weeks, demonstrating that miR-146a-/- mice develop insulin resistance following DIO (Fig 2e). Taken together, these data show that miR-146a is required in mice to prevent development of a T2D phenotype during DIO.

### miR-146a constrains inflammation in metabolic tissues during NCD and HFD

**Fig 3:**
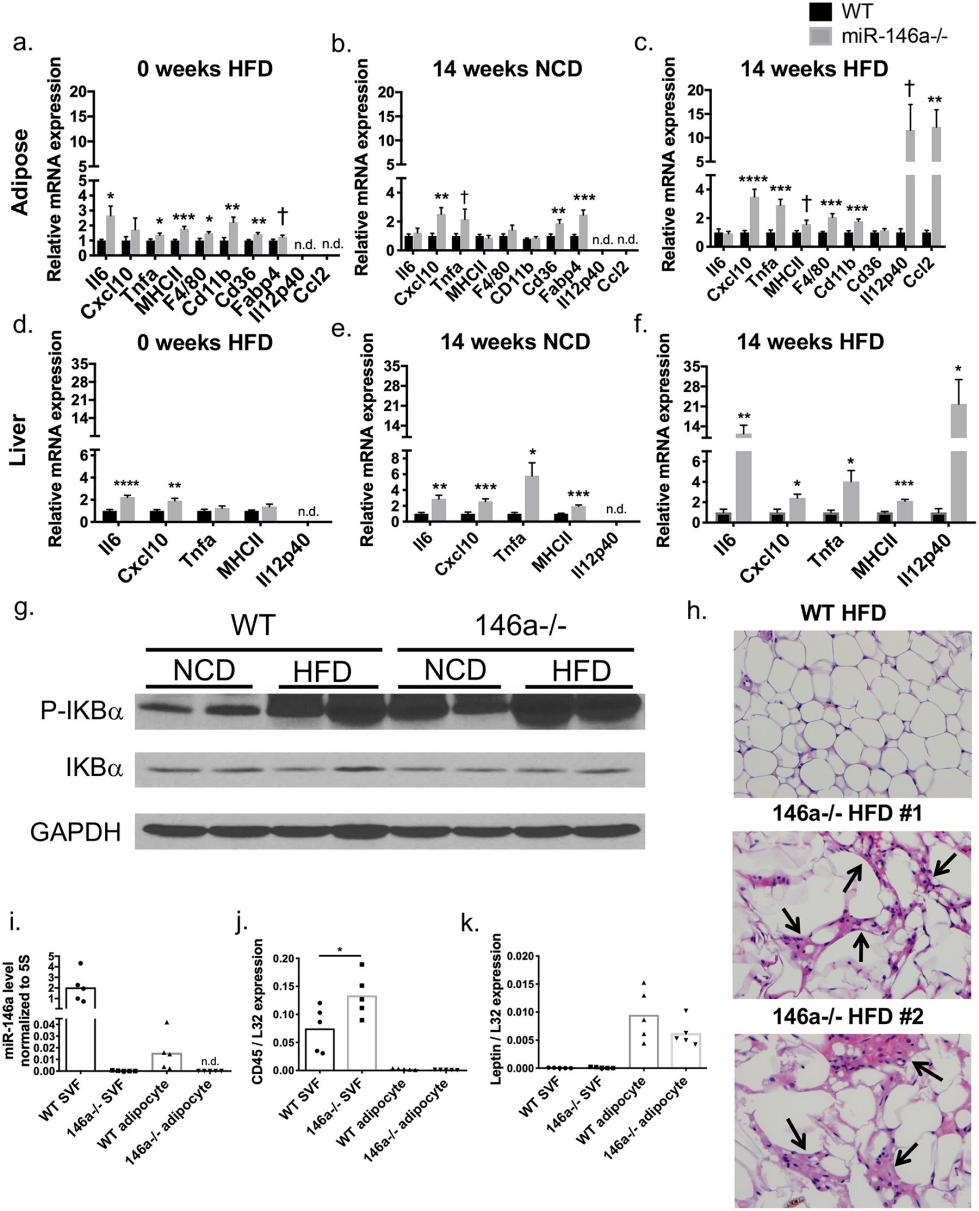
miR-146a constrains inflammation and is expressed in adipose tissue. (A-C) qRT-PCR expression levels of various mRNAs (shown on x axis) measured in WT (black) or miR-146a (grey) mice in whole VAT at (A) 0 weeks HFD; (B) 14 weeks on NCD; and (C) 14 weeks on HFD. (D-F) qRT-PCR expression levels of various mRNAs (shown on x axis) measured in WT or miR-146a mice in liver at (D) 0 weeks HFD; (E) 14 weeks on NCD; and (F) 14 weeks on HFD. (G) Western blot for P-IKBa, total IKBa, and GAPDH in lysates of whole VAT collected from 20-week old WT or miR-146a-/- mice fed NCD or HFD. (H) H&E staining of VAT of WT and miR-146a-/- mice fed HFD for 18 weeks. Black arrows indicate areas of inflammation. Two representative examples from miR-146a-/- VAT are shown, with one representative WT mouse; 400x magnification. (I) Mature miR-146a expression relative to 5s rRNA, measured in the SVF and adipocyte fraction of WT and miR-146a-/- mice fed NCD. (J-K) *Ptprc* (CD45) and Leptin expression levels, relative to *L32*, were measured as controls for fractionation purity. Samples were normalized by setting WT expression to 1. Data are shown as mean ± SEM (a-f) (n = 10) or as individual mice (h-k) (n = 5); lysates from individual mice (g) (n = 2). p-value was calculated using two-tailed Student’s t-test. *p<0.05; **p<0.01; ***p<0.001; ns = not significant. See also S4 Fig.

miR-146a has previously been shown to reduce inflammation in several contexts [7,14]; therefore, we hypothesized that it may regulate inflammatory gene expression within metabolic tissues, including VAT and liver. To determine whether gene expression changes were due to HFD, inflammatory gene expression was measured via qPCR in VAT and liver tissue from WT and miR-146a-/- mice on NCD or HFD. Prior to HFD, miR-146a-/- mice had increased pro-inflammatory gene expression in VAT (Fig 3a) and liver (Fig 3d) compared to WT controls. These differences were also observed in miR-146a-/- controls on NCD for 14 weeks, where inflammatory and fatty acid transporter genes were higher in miR-146a-/- adipose tissue (Fig 3b) and liver (Fig 3e). In miR-146a-/- mice fed HFD for 14 weeks, we observed greater increases in a variety of inflammatory genes (Fig 3c and 3f) from miR-146a-/- compared with WT mice. Additionally, VAT from miR-146a-/- mice had increased NFκB activation, as measured by phosphorylated IKBα which serves as a surrogate for measuring NF-κB activation [23], and this was further increased by HFD (Fig 3g). These data indicate that inflammation in miR-146a-/- metabolic tissues occurs in the absence of HFD and likely predisposes mice to obesity and glucose dysregulation in response to elevated fat in the diet. DIO further activates inflammation in miR-146a-/- mice, as the fold change in pro-inflammatory gene expression was further increased during HFD.

Blinded histological examination of H&E-stained VAT revealed greater accumulation of inflammatory foci resembling crown-like structures in miR-146a-/- than in WT mice following HFD (Fig 3h), indicating increased immune cell activation in VAT during DIO. miR-146a has known roles within immune cells, and immune cells in VAT cause inflammation that drives metabolic disease; thus, we hypothesized that the absence of miR-146a from immune cells may exacerbates metabolic disease. To assess expression of miR-146a within certain adipose-associated cell types, we separated the SVF, which contains ATMs, fibroblasts, preadipocytes, and other leukocytes, from the adipocyte fraction of WT and miR-146a-/- VAT. qPCR was performed on RNA from these fractions, revealing mature miR-146a more highly expressed in SVF than in the adipocyte fraction of WT mice as hypothesized (Fig 3i). As a control, we measured mature miR-146a in the SVF and adipocyte fractions of miR-146a-/- samples where it was not detected (Fig 3i). Specific expression of *Ptprc* (CD45) in the SVF and *Leptin* in the adipocyte fraction ensured proper separation of these fractions (Fig 3j and 3k). These results indicate that miR-146a is more abundant within the SVF of VAT than adipocytes, suggesting that miR-146a exerts its regulatory function within adipose tissue immune cells. However, miR-146a might also be expressed and function in adipocyte progenitors or other non-immune cells and this could also contribute to the observed phenotypes.

### Exaggerated obesity and hyperglycemia in miR-146a-/- mice is not miR-155 dependent

We previously showed that some age-related chronic inflammatory phenotypes exhibited by loss of miR-146a are dependent upon miR-155, an inflammation-promoting miRNA [8, 10] whose deletion in mice has been shown to reduce DIO [24]. We thus wanted to assess whether the metabolic disease and inflammatory phenotypes in miR-146a-/- mice during DIO were also dependent on miR-155. To test this, we fed HFD to WT, miR-155-/-, miR-146a-/-, and miR-155-/- miR-146a-/- (double knockout or DKO) mice for a period of 12 weeks and measured weight gain over time. miR-155-/- and WT mice gained similar weight in response to HFD, while miR-146a-/- and DKO mice both gained significantly more weight than either WT or miR-155-/- (S4a and S4b Fig). Fasting blood glucose followed this pattern, with DKO and 146a-/- mice having similar levels to each other, both higher than WT or miR-155-/- (S4c Fig). Further, miR-146a-/- and DKO mice had larger VAT pads (S4d Fig), a higher percent body fat (S4e Fig), and reduced lean body composition (S4f Fig) compared to WT or miR-155-/- mice. As evidenced by DKO mice showing no significant differences from the miR-146a-/- mice, these results demonstrate that miR-155 does not play a significant role in driving the obesity and metabolic disease phenotypes observed in miR-146a-/- mice on HFD.

### Enhanced inflammatory and mTOR/AKT gene expression by ATMs from miR-146a-/- mice on HFD

**Fig 4:**
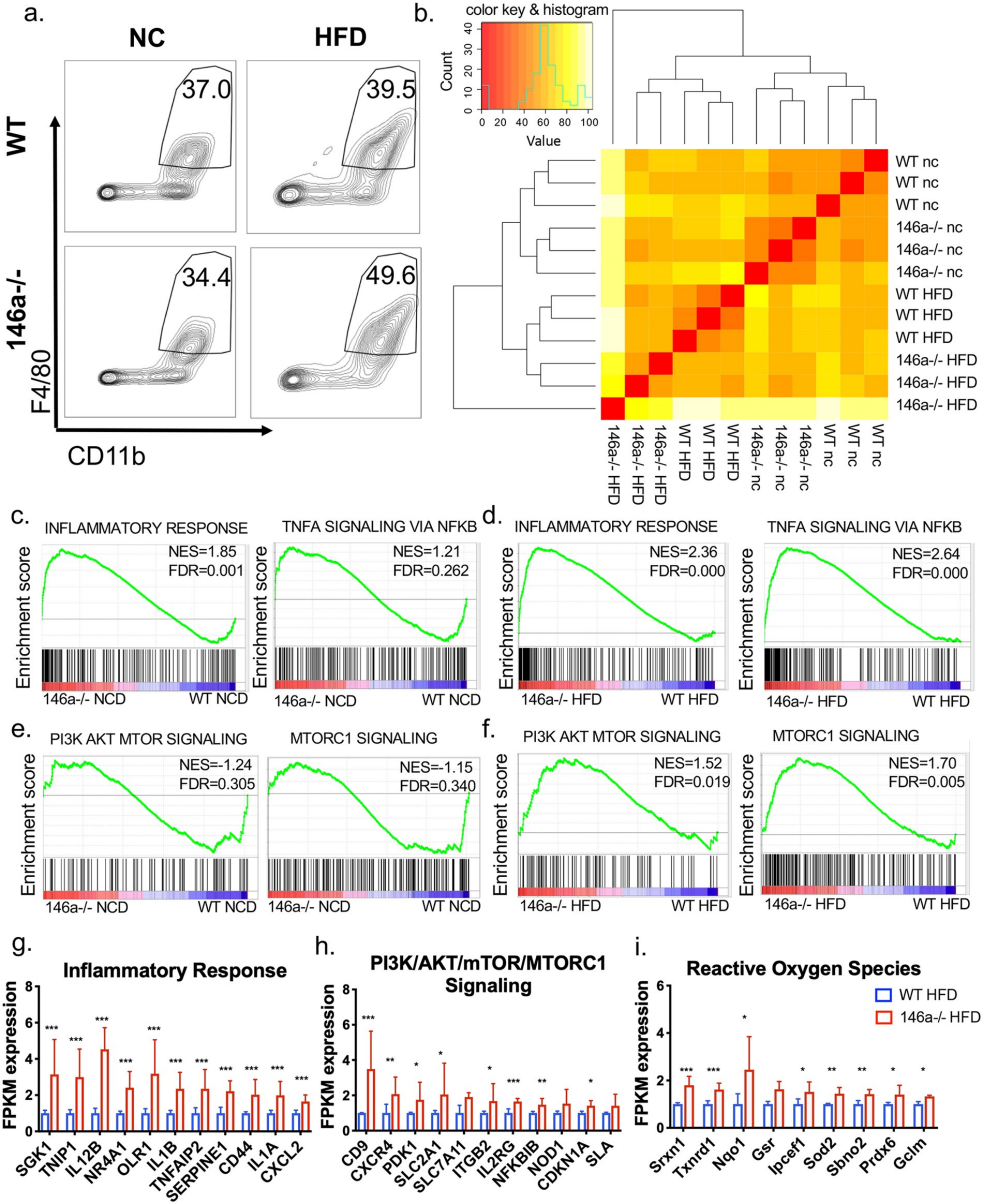
miR-146a regulates adipose tissue macrophage gene expression during NCD and HFD. (A) Representative FACS plots of cells collected from the SVF of 20-week old WT and miR-146a-/- mice fed HFD or NCD. Cells were sorted for CD11b+ F4/80+ markers from live, singlet CD45+ cells. Numbers indicate percent cells in gate. (B) Heat map showing total gene expression of sorted ATMs from WT and miR-146a-/- mice fed NCD or HFD, as measured by RNA-seq. Red indicates 100% similarity in total RNA expression to compared sample; yellow indicates dissimilarity. (C-F) Gene Set Enrichment Analysis plots of miR-146a-/- (red) and WT (blue) mice showing (C) (left) enrichment of the ‘inflammatory response’ in miR-146a-/- mice during NCD, and (right) no statistical enrichment of ‘TNFa signaling via NFkB’ during NCD; (D) enrichment of the ‘inflammatory response’ and ‘TNFa signaling via NFkB’ in miR-146a-/- mice during HFD; (E) no statistical enrichment of ‘PI3K/AKT/mTOR signaling’ or ‘mTORC1 signaling’ during NCD; (F) enrichment of ‘PI3K/AKT/mTOR signaling’ and ‘mTORC1 signaling’ in miR-146a-/- mice during HFD. (g-i) Relative expression of the top hits within (G) the ‘inflammatory response’, (H) ‘PI3K/AKT/mTOR/mTORC1 signaling’, and (I) ‘Reactive oxygen species’ upregulated in miR-146a-/- ATMs compared to WT, as determined by RNA-seq. The WT FPKM averages were set to a relative expression value of 1, with data shown as mean±SD. p-value was calculated using two-tailed Student’s t-test. *p<0.05; **p<0.01; ***p<0.001; ns = not significant. NES = normalized enrichment score; FDR = false discovery rate, where FDR<0.25 is statistically significant. See also S5 Fig.

Because macrophage hallmark genes are at higher levels in VAT of miR-146a-/- mice fed HFD (Fig 3a–3c and 3h), and macrophages have been shown to regulate metabolic disease in adipose tissue [25], we next determined which genes and pathways are affected by miR-146a in ATMs. To identify the genes regulated by miR-146a in adipose tissue *in vivo*, we isolated ATMs from adult WT and miR-146a-/- mice fed NCD or HFD. VAT was digested, and CD45+ CD11b+ F4/80+ cells were sorted using fluorescence activated cell sorting (FACS). A comparable proportion of macrophages was sorted from NCD animals of both genotypes, while a trending increase in ATM percentage was measured in miR-146a-/- mice on HFD compared to WT controls (Fig 4a and S5a Fig). The total number of macrophages sorted was marginally increased in miR-146a-/- compared to WT mice on NCD, but not different between mice on HFD (S5b Fig). We also measured VAT B and T cells and saw no difference between the genotypes (S5c Fig).

Next, RNA-sequencing using ATM RNA was performed and we saw that gene expression clustered by genotypes and diet treatments, indicating that genes are regulated by miR-146a on its own and in combination with specific diets (Fig 4b). To determine the types of genes regulated by miR-146a in ATMs during NCD and HFD we performed both an Ingenuity Pathway Analysis and a Gene Set Enrichment Analysis using our ATM RNA-Seq data. Among significant hits for gene sets enriched in miR-146a-/- macrophages were inflammatory pathways including interferon gamma and alpha, glycolysis and hypoxia, IL6/Jak/Stat3 signaling, and complement (S5d and S5e Fig). Of note, the top two pathways enriched in miR-146a-/- HFD macrophages were ‘TNFa signaling via NFkB’ and ‘Inflammatory Response’ (Fig 4d and S5d Fig); interestingly, ‘Inflammatory Response’ was also enhanced in miR-146a-/- NCD cells (Fig 4c and S5e Fig). Inflammatory response genes upregulated in miR-146a-/- HFD ATMs (Fig 4g) indicate that miR-146a regulates inflammatory ATM pathways in both lean (NCD) and obese (HFD) adipose tissue, corroborating our finding that inflammatory cytokine expression is elevated in miR-146a-/- mice regardless of diet.

The PI3K/AKT/mTOR and mTORC1 signaling gene sets were enriched in miR-146a-/- HFD macrophages, which was not seen in ATMs from mice on NCD (Fig 4e and 4f and S5d Fig). Genes from these enriched gene sets (Fig 4h) indicate a novel role for miR-146a in regulating the mTOR/AKT pathway which has previously been linked to cellular metabolism and obesity. Interestingly, the Reactive Oxygen Species gene set was upregulated in miR-146a-/- macrophages during HFD (Fig 4i and S5d Fig). Increased reactive oxygen species are important for effective inflammatory responses and are produced in LPS-activated macrophages which undergo a metabolic switch to downregulate oxidative phosphorylation and increase aerobic glycolysis [26]. Consistent with this, the glycolysis gene set was also enriched (S5d Fig). Altogether, these data suggest that miR-146a regulates gene expression in ATMs, particularly of the inflammatory response as well as a unique subset of metabolic pathways during DIO.

### miR-146a targets Traf6 to regulate macrophage inflammation and metabolism

**Fig 5:**
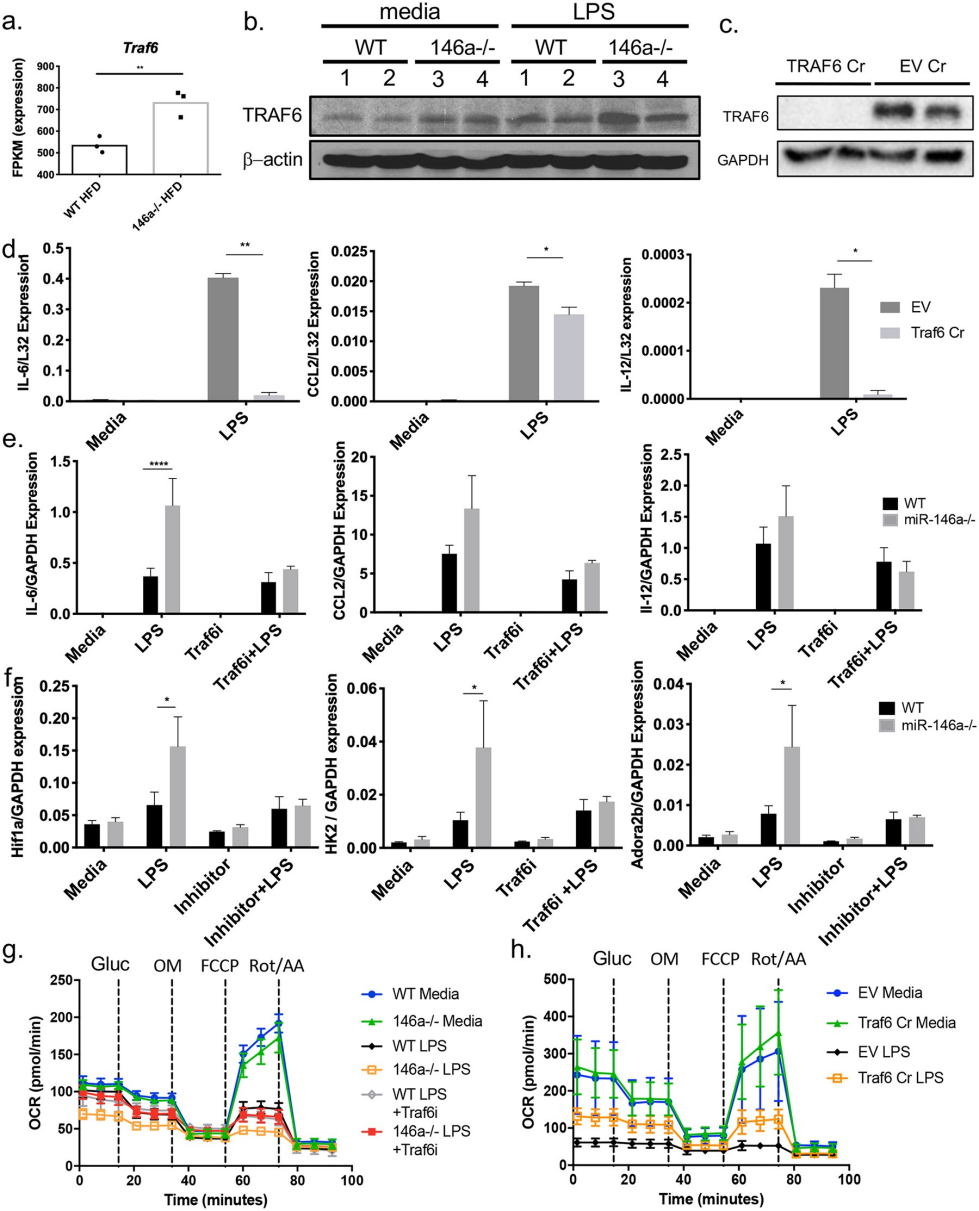
The miR-146a target Traf6 downregulates inflammation and alters cellular metabolism in activated macrophages. (A) FPKM values of Targetscan-predicted miR-146a-5p target *Traf6* in WT or miR-146a-/- ATMs during HFD, as determined by RNA-seq. (B) Western blot for Traf6 and beta-actin in lysates from WT (1 and 2) or miR-146a-/- (3 and 4) BMDMs double-stimulated with media control or LPS. (C) Western blot for Traf6 and GAPDH in lysates from Traf6 CRISPR and Empty Vector control RAW cells stimulated with media control or LPS. (D) mRNA expression of *il6*, *ccl2*, and *il12* in Traf6 Cr and EV (empty vector) control RAW cells. (E) mRNA expression of *Il6*, *Ccl2*, and *Il12* in WT or miR-146a-/- BMDMs treated with media, LPS, Traf6i, or Traf6i+LPS. (F) mRNA expression of metabolic genes *Hif1a*, *Hk2*, and *Adora2b* in WT or miR-146a-/- BMDMs double stimulated with media, LPS, Traf6i, or Traf6i+LPS. (G) OCR of WT and miR-146a-/- BMDMs double-stimulated with media, LPS, Traf6i, or Traf6i+LPS, followed by Seahorse analysis with addition of glucose (gluc), oligomycin (OM), FCCP, and rotenone (rot)/antimycin A (AA). (H) OCR of Traf6 Cr and EV control RAW cells double-stimulated with LPS or media, followed by addition of glucose (gluc), OM, FCCP, and Rot/AA. Data are shown as mean±SEM (d-f) or as individual mice (a-b). p-values were calculated using two-tailed Student’s t-test. *p<0.05; **p<0.01; ***p<0.001; ****p<0.0001.

To further understand the mechanism by which miR-146a regulates metabolism and inflammation in ATMs, we analyzed the expression levels of predicted miR-146a targets from Targetscan in our RNA-seq dataset. Among the relevant predicted targets, *Traf6* mRNA expression was significantly elevated in the miR-146a-/- HFD ATMs compared to WT (Fig 5a). Traf6 is a *bona fide* miR-146a target in several inflammatory contexts [7, 27] making it a viable option for further analysis in our system. Traf6 protein levels were increased in miR-146a-/- bone marrow-derived macrophages (BMDMs) stimulated with LPS compared to WT controls (Fig 5b). To further investigate Traf6 as the miR-146a target regulating inflammation in our model, we developed Traf6 mutant Raw264.7 macrophages using Crispr-Cas9 (Traf6 Cr) to disrupt expression of this protein (Fig 5c). Upon deletion of Traf6, we observed reduced LPS-mediated inflammatory cytokine expression, consistent with miR-146a dampening these inflammatory genes by downregulating Traf6 (Fig 5d). Additionally, we used a previously characterized Traf6 peptide inhibitor (Traf6i) to reduce Traf6 activity in primary miR-146a-/- BMDMs and observed reduced inflammatory gene expression in response to LPS (Fig 5e) [28]. These data support a model whereby miR-146a regulates adipose inflammation at least in part by targeting Traf6 in ATMs.

RNA-seq also suggested an altered metabolic state in miR-146a-/- ATMs specifically when placed on HFD. This was indicated by increases in gene sets for PI3K/AKT/mTOR signaling, glycolysis, and reactive oxygen species (Fig 4f and 4h–4i and S5d Fig). Therefore, we examined the role of miR-146a in regulating metabolism in activated BMDMs. First, we assessed expression of glycolysis genes including HK2 and HIF1a and found that they were induced to higher levels in LPS-treated miR-146a-/- vs. WT control BMDMs, but not when miR-146a-/- BMDMs were treated with a Traf6i (Fig 5f). We also measured oxidative phosphorylation in LPS-activated miR-146a-/- BMDMs. Throughout a Mito Stress Test, miR-146a-/- BMDMs stimulated with LPS had low oxygen consumption rates (OCR) as compared with WT controls and media-treated cells, indicating lower oxidative respiration and little responsiveness to added glucose or to mitochondrial respiration inhibitors oligomycin (OM), FCCP, or Rotenone/Antimycin A (Fig 5g). Additionally, Traf6 mutant cells exhibited greater OCR when compared with WT empty vector controls (Fig 5h). These data are consistent with previous studies showing that pro-inflammatory M1 macrophages undergo a metabolic switch involving increased aerobic glycolysis and decreased oxidative phosphorylation. These results suggest a role for miR-146a in restricting this metabolic alteration in activated macrophages. Altogether, these data suggest that miR-146a regulates macrophage activation by influencing both inflammatory response genes and metabolic state through targeting of Traf6.

### Inhibition of mTOR with rapamycin reduces the excessive weight gain by miR-146a-/- mice on HFD

**Fig 6:**
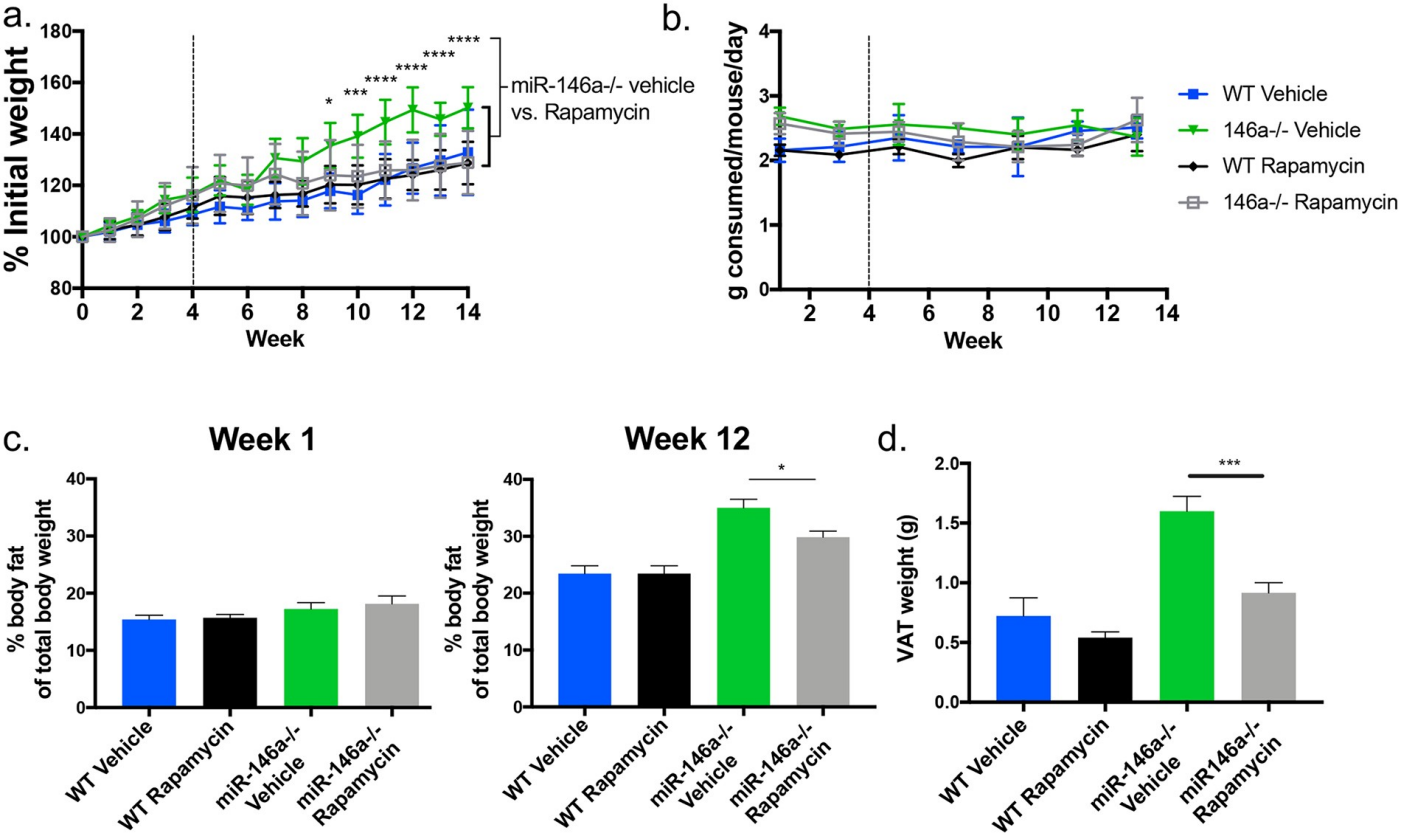
mTOR inhibition reduces the miR-146a HFD phenotype *in vivo*. (A) C57BL/6 WT and miR-146a-/- mice were placed on HFD for 4 weeks. After 4 weeks, mice were regularly injected with either rapamycin or vehicle control for the duration of the HFD. Shown is the measure of percent weight over time, based on initial weight. Significance levels are indicated for miR-146a-/- vehicle vs. rapamycin groups. (B) Amount of chow consumed per mouse per day over time of diet. (C) % fat of total body mass at week 1 and week 12 HFD. (D) Weight of WT and miR-146a-/- VAT after 14 weeks of HFD in rapamycin or vehicle groups (n = 8/group). p-values for (A) were calculated using RM-ANOVA, corrected for multiple comparisons using Tukey. p-values for (C-D) were calculated using a two-tailed Student’s t-test *p<0.05; **p<0.01; ***p<0.001; ****p<0.0001.

Having identified a novel role for miR-146a in regulating macrophage metabolism, including evidence for increased activation of the Akt/mTOR pathway, we tested whether administration of rapamycin, an inhibitor of mTOR activation, would reverse the miR-146a-/- DIO phenotypes. WT and miR-146a-/- mice on HFD were injected intraperitoneally with rapamycin 3 times per week for the last 10 weeks of a 14 week HFD experiment. Consistent with a role for elevated mTOR as a driver of DIO, miR-146a-/- mice given rapamycin put on significantly less weight than miR-146a-/- mice given the vehicle control, and resembled WT control mice in terms of their weight, percent body fat and VAT pad size (Fig 6a, 6c and 6d), with no differences in food consumption (Fig 6b). These results support a model whereby increased mTOR activation in miR-146a-/- macrophages, and possibly other cell types, supports increased adiposity in response to a high fat diet.

## Discussion

Obesity is a global problem, with an estimated 600 million individuals affected worldwide [29]. Understanding its pathogenesis is crucial for developing therapies and finding a cure. Our study demonstrates a physiologically relevant role for miR-146a in this context, where miR-146a is required for protection from obesity and metabolic disease during HFD and loss of miR-146a causes underlying inflammation that acts as a predisposing factor for DIO. This underlying inflammation, however, does not trigger metabolic disease on its own in our model. Although we cannot rule out a possible role for miR-146a in regulating certain aspects of metabolism in the presence of a normal diet, most metabolic phenotypes analyzed in this study only showed robust differences in the context of HFD. Thus, the combination of a miR-146a deficiency together with HFD triggers the onset of obesity and metabolic disease. This is likely caused by pro-inflammatory adipokines expressed during caloric excess that act in concert with inflammatory pathways to signal a need for increased fat storage. Importantly, our results suggest that obesity onset can be driven by a combination of miRNA genetics and diet, a finding which could have clinical relevance.

miR-146a is highly expressed within the SVF of adipose tissue which is rich in leukocytes, suggesting a role for this miRNA within immune cells. Based on the miR-155 data presented above, we speculate that our miR-146a-/- mouse obesity and metabolic disease phenotype might be T cell-independent as our previous model revealed a T cell-intrinsic role for miR155 during age-dependent chronic inflammation or antitumor immunity upon loss of miR-146a [8, 10]. Rather, miR-146a has been shown in previous studies to be important within myeloid cells [7], and our findings suggest that ATMs are at least one cell type where this miRNA may act to protect against inflammation and DIO. However, other cell types also express miR-146a and could play a role, such as B or T lymphocytes or preadipocytes, which are also found in the SVF. miR-146a has been shown to function in each of these cell types in other contexts [8, 10–12], and future work utilizing conditional knockout animals to characterize the cell-specific roles of miR-146a will further contribute to our understanding of miR-146a’s role in obesity development and progression.

miR-146a is well-known for regulating genes that are part of the inflammatory response, which we have shown to be dysregulated during DIO; however, other regulatory functions of miR-146a are not well-studied and could be key to understanding its role in disease as well as its therapeutic potential. Here, we found that miR-146a regulates the metabolism of activated macrophages, a previously unrecognized function for miR-146a. We saw that activated miR-146a-/- macrophages undergo metabolic reprogramming characterized by decreased oxidative phosphorylation, and this phenotype could be rescued in miR-146-/- macrophages using a Traf6 peptide inhibitor and was the opposite in Traf6-deficient macrophages. In addition to decreased OCRs observed in miR-146a-/- macrophages, the glycolysis gene set was significantly enriched in our RNA sequencing data, and we saw increased expression of glycolysis genes by qPCR which could again be rescued with the Traf6 inhibitor. This suggests that miR-146a represses Traf6 not only to control inflammatory gene expression, but also to limit the switch from oxidative phosphorylation to glycolytic metabolism during inflammation [30, 31]. Our GSEA also indicated increased mTOR activation, which has been shown to regulate both oxidative phosphorylation and glycolysis [32]. Of significance, we found that blocking mTOR *in vivo* using rapamycin could reverse the increased adiposity observed in miR-146a-/- mice on HFD.

Although miR-146a has been shown to have many direct targets, we found Traf6 to be de-repressed in miR-146a-/- ATMs. There is evidence that Traf6 is involved in activating Akt and mTOR [33, 34] which may explain how these pathways are impacted by miR-146a. This may also be a more complex mechanism, as Traf6 itself localizes to the mitochondria during TLR activation, a process that is required for activation of inflammation and ROS production by macrophages [35]. Further work will be needed to understand the pathways and mechanisms through which miR-146a and Traf6 regulate macrophage metabolism during DIO.

Altogether, our results point to a physiologically relevant role for miR-146a in protecting against DIO. Recently, direct targeting of miRNAs has been shown to have therapeutic success in disease treatment [36], and miRNA mimics are currently being investigated for their therapeutic potential [37]. Human studies have shown that miR-146a expression is decreased in individuals with obesity and T2D [15, 16]. This observation, combined with our current study, suggests that utilizing miRNA mimics to enhance miR-146a levels may have therapeutic potential for treating obesity, diabetes, and other forms of metabolic inflammation and disease [15]. Overall, our study provides evidence that the combination of diet and miRNA genetics can have a substantial impact on obesity and diabetic phenotypes in mammals.

## Materials and methods

### Ethics statement

Experimental procedures were performed with the approval of the Institutional Animal Care and Use Committee (IACUC) of University of Utah, USA, approval number: 17–03006.

### Sample size

Group sizes of five to ten mice were used consistent with previous publications carrying out similar experiments.

### Rules for stopping data collection

Data collection was stopped according to previous studies, which measure DIO over the course of several months. Experiments were carried out beyond preliminary results from initial experiments, which showed that miR-146a-/- mice develop statistically significant weight changes and other metabolic differences after 5–6 weeks of HFD.

### Data inclusion/exclusion criteria

Data were included given that experiments were carried out accurately according to the design. Mice were excluded from studies if they showed signs of illness or dishevelment during experimentation; however, none of these issues were observed.

### Outliers

Outliers were removed according to the “identify outliers” tool on Graphpad Prism 7 software, in which data points at least two standard deviations from the mean are removed. Outliers removed in the study have been reported.

### Selection of endpoints

Endpoints for HFD were selected prospectively, in that data were collected from mice before treatment, during an early timepoint, and following induction of obesity. Diabetes and other metabolic measurements were taken following induction of DIO at later timepoints of disease.

### Replicates

HFD experiments were performed in female mice four times, each with at least five experimental replicates to account for biological variability. Additionally, experiments were performed in male mice to account for possible sex differences. Tissues were collected from mice for various downstream assays in three of the four experiments with females. *In vitro* studies were performed at least 2 independent times, or utilized cells from multiple mice per group.

### Research objectives

The purpose of this study was to determine the role of miR-146a during diet induced obesity, diabetes, and metabolic inflammation. Before experiments, we hypothesized that feeding miR-146a-/- mice HFD will result in increased obesity and diabetic phenotypes compared with WT controls because of miR-146a’s anti-inflammatory regulation of NF-kB, leading to increased inflammation within metabolic and immunologic tissues.

### Research subjects

Experiments were performed on C57BL6 miR-146a-/- and WT mice described previously [11]. Additionally, BMDMs derived from WT and miR-146a-/- mice and the macrophage cell line Raw264.7 were utilized for *in vitro* work.

### Experimental design

Studies performed were controlled laboratory experiments. WT and miR-146a-/- mice were fed NCD or HFD for up to 18 weeks and weighed throughout the experiment. Food consumption was measured each week. Body composition and other metabolic parameters were measured using NMR body composition analysis and CLAMS metabolic cages. Fasting blood glucose was measured by fasting mice for 6 hours and reading blood glucose levels. Glucose tolerance tests were performed by injecting mice with glucose and measuring blood glucose levels at timepoints over 120 minutes. After up to 18 weeks on HFD, mice were sacrificed and tissues collected. Measurements included RNA and protein quantification from liver and VAT. WT and miR-146a-/- BMDMs macrophages were cultured, stimulated with LPS, and collected for RNA and protein levels. Additionally, mito-stress Seahorse tests were performed on LPS-stimulated macrophages.

### Randomization

Mice were age and sex-matched between WT and miR-146a-/-. Mice were randomly selected from various breeder pairs, and multiple experimental repeats were performed over the course of several years on both males and females to account for environmental differences. Mice were also purchased from Jackson Laboratories and HFD experiments repeated to account for possible varied animal facility environments.

### Blinding

HFD experiments were not conducted in a blinded manner, but data were analyzed to ensure there was a blinded assessment of outcomes. Of note, measurements such as weight gain are quantitative and therefore limit data collection bias. The investigators were aware which mice were WT and miR-146a-/- mice, as the animal facility requires genotypes to be posted on cages. Histological sections of tissue were scored blindly by a trained pathologist (Mary Bronner).

### Mice

All WT and miR-146a-/- mice were on a C57BL/6 background [11] and were bred and housed in a specific pathogen-free mouse facility at the University of Utah, USA. WT (strain 000664) and miR-146a-/- (strain 016239) mice were also purchased from Jackson Labs to ensure phenotypes were repeatable in mice exposed to different housing. Mouse experiments were performed in female and male mice starting at 6 weeks, with results showing representative data from female cohorts. Mice were euthanized using carbon dioxide chambers at experimental endpoints.

### Cell culture

Cells from the RAW 264.7 murine macrophage cell line were cultured in DMEM complete media and kept at 37°C with 5% CO_2_. Cells were passaged every 2–3 days to maintain logarithmic growth.

### Primary culture

Bone marrow was isolated from WT or miR-146a-/- mice, RBC lysed, and plated in DMEM complete media with 20 ng/mL mouse MCSF (Biolegend). At 4 days of culture, fresh media containing MCSF was added to the cells. At day 7, cells were stimulated with 1 μg/mL LPS (Sigma) for 24 hours. At 24-hours, media was removed and cells were stimulated with a second hit of fresh media containing LPS for an additional 2–6 hours. For Traf6i experiments, cells were pre-incubated with 20 μM Traf6i for 1 hour prior to each LPS stimulation. Protein lysate or RNA was collected using Qiazol/miRNeasy kit (Qiagen) and Western or qRT-PCR was performed on these cells.

### Diet-induced obesity and metabolic analysis

Mice were placed on NCD (10% kcal fat; Research Diets D12450Bi) or HFD (45% kcal fat; Research Diets D12451i) starting at 4–6 weeks of age for 12 to 18 weeks. Mice were weighed weekly, and food consumption was tracked. Body composition, including body fat, lean mass, and fluid, was measured via TD-NMR using the Bruker Minispec Body Composition Analyzer. Energy expenditure, movement, VO_2_ max, VCO_2_ max, respiratory exchange ratio (RER), and food and drink consumption were measured using CLAMS metabolic cages, which was performed by the Metabolic Phenotyping Core. Serum was collected from mice treated with HFD for 0, 2 or 17 weeks and Leptin levels were measured via MAGPIX, using the mouse metabolic hormone panel (Millipore). For the rapamycin experiments, mice were placed on HFD for four weeks prior to administration of rapamycin, and then kept on HFD and intraperitoneally (i.p.) injected with rapamycin (LC Laboratories) three times per week for 10 weeks. Rapamycin was prepared in a solution of 100% ethanol, 10% PEG400, and 10% Tween80 and was injected at 4 mg/kg based on body weight.

### Glucose and insulin analysis

For glucose tolerance tests (GTT), mice were fasted for 6 hours prior to experimentation. 100 mg/mL of D-(+)-Glucose (Sigma) was prepared for intraperitoneal injection into mice and administered at 1mg/g based on body weight. Blood glucose levels were measured at timepoints 0 (prior to injection), 5, 15, 30, 60, and 120 minutes following injection using a Bayer Contour glucometer. Blood for this experiment was taken via tail nicks. For insulin ELISA, serum was collected from 6-hour fasted mice, and insulin was measured using a mouse insulin ELISA kit (Crystal Chem). The homeostasis model of β-cell function (HOMA-B), the β-cell function index, was calculated using the formula: HOMA-B = [insulin (microunits per milliliter)20]/[glucose (millimoles per liter) − 3.5] and homeostasis model assessment insulin resistance index (HOMA-IR), the insulin resistance index, was calculated using the formula: HOMA-IR = [glucose (millimoles per liter)insulin (microunits per milliliter)/22.5]. Both HOMA-B and HOMA-IR were calculated using fasting values [38].

### Adipose and liver gene expression and protein analysis

Approximately 0.1 grams of VAT or liver was collected from mice. RNA was extracted from these tissues using miRNeasy kits (Qiagen), cDNA was made using qScript cDNA Synthesis Kit (Quanta), and GoTaq Master Mix (Promega); The LC480 (Roche) was used for qPCR to measure expression of various genes. Primer set sequences can be found in supplemental materials and methods. For Western blots, levels of phosphorylated IKBα (Cell Signaling), IKBα (Cell Signaling), Traf6 (Abcam), beta actin (Abcam), and GAPDH (Santa Cruz) were measured from whole VAT lysates or BMDMs from WT or miR-146a-/- mice.

### Adipocyte and stromal vascular fraction separation

Reproductive VAT pads were removed from mice, minced, and digested in buffer containing HBSS, Collagenase D (Roche), and Dispase (Worthington) for 1 hour at 37 degrees. Homogenates were placed on ice for 30 minutes then spun to separate adipocytes from SVF. Adipocytes, which float on the top, were considered the adipocyte fraction. Supernatant was then removed to obtain the pelleted SVF. RBC lysis buffer was added to the SVF, which was spun and washed. RNA was collected from the adipocyte and SVF fractions via Qiazol/miRNeasy Kit (Qiagen), and miR-146a levels were measured using miRCURY LNA RT PCR (Exiqon) and the mmu-miR-146a primer from Exiqon. Primer sequences for CD45 and Leptin can be found in supplemental materials and methods.

### RNA-seq using adipose tissue macrophages

WT and miR-146a-/- mice were treated with NCD (Teklad 2920, Envigo) or HFD from 6 weeks of age (Research Diets D12451i) and were sacrificed at 20 weeks old. SVF pellets from three mice were combined to obtain sufficient cells for sorting and RNA collection for RNA-Seq that was performed as described above. Cells were stained with antibodies to CD45, CD11b, and F4/80, and live, singlet, CD45+ CD11b+ F4/80+ cells were sorted using a FACS Aria (BD). RNA was collected via Qiazol/RNeasy Kit (Qiagen). Library preparation used Illumina TruSeq Stranded RNA Kit with Ribo-Zero Gold and RNA-seq was performed using Illumina HiSeq 50 cycle single-read sequencing version 4. Sequence alignment was performed through the University of Utah Bioinformatics Core Facility and Geneset Enrichment Analysis (GSEA) and Ingenuity Pathway Analysis software were used to examine types of genes that were up or downregulated in miR-146a-/- macrophages. Predicted miR-146a targets were obtained via Targetscan for mmu-miR-146a-5p. The RNA-Seq data have been deposited into NCBI GEO under accession number GSE119703.

### lentiCRISPR infection

RAW 264.7 cells were infected with a lentiviral CRISPR construct containing a Traf6 guide RNA or an Empty Vector using the same system we have previously described [39]. The two Traf6 guide RNA constructs were: GGAGATCCAGGGCTACGATG and GATGGAACTGAGACATCTCG. For infection, the lentiCRISPR and packaging plasmids pVSVg and psPAX2 were transfected into 293T cells using Trans-IT to produce virus. The lentiCRISPR was then transduced into RAW 264.7 cells via spin infection for 90 minutes at 30 C. The day after spin infection, cells were selected with 3.75 ug/mL puromycin. Media plus puromycin was replaced every 3 days and cells split as needed. Cells were allowed to grow for 17 days before experimental use.

### Seahorse metabolic analysis

WT and miR-146a-/- BMDMs were cultured in DMEM and mMCSF for 7 days, then seeded into a 96-well Seahorse XF-96 plate. Cells were treated with LPS or media control and then incubated for 24 hours, followed by a second stimulation of LPS. For Traf6i experiment, cells were pre-incubated with 20 μM Traf6i or media control for 1 hour prior to each LPS stimulation. 1 hour after the second LPS stimulation, the Seahorse XF Mito Stress test was performed using a Seahorse XF-96 analyzer. Alternatively, Cultured RAW 264.7 cells were seeded into a 96-well Seahorse XF-96 plate and LPS-stimulated at 24 hours and 1 hour prior to the Seahorse experiment. This assay was performed by the Metabolic Phenotyping Core Facility at the University of Utah, USA. Concentrations of the following were added into the injection ports: 25 mM glucose (A), 1.5 μM oligomycin A (B), 1.5 μM FCCP + 1 mM sodium pyruvate (C), 2.5 μM antimycin A + 1.25 μM rotenone (D). Glucose and pyruvate-free assay media were used during the Seahorse assay, and cultured BMDMs or RAW 264.7 cells were washed with assay media before beginning the test.

### Quantification and statistical analysis

Graphpad Prism 7 software was used for graphing and statistical analysis of experimental data. Two-tailed Student’s T tests were used to calculate p-values. Quantitative data are displayed as mean +/− SEM. P-values are shown as indicated: *≤0.05, **≤0.01, ***≤0.001, ns p>0.05. For RNA-Sequencing, Ingenuity Pathway Analysis and Gene Set Enrichment Analysis were performed. FDR values are shown in GSEA plots, where FDR<0.25 is statistically significant.

## Supporting information

S1 Fig miR-146a is required to prevent weight gain and Leptin accumulation during HFD. (A) Each line shows average percent weight gain over time during an individual experimental repeat of young female WT (blue) and miR-146a-/- (red) mice on HFD. (B) Percent weight gain over time of WT and miR-146a-/- mice on NCD. (C) Body weight gain (in grams) over time of WT and miR-146a-/- mice on NCD. (D) TD-NMR body composition measurements showing percent body fat of WT and miR-146a-/- mice at 5, 8, and 12 weeks of NCD. (E) Leptin protein levels from serum of 6-hour fasted WT and miR-146a-/- mice at 2 and 17 weeks HFD. (F) Comparison of percent weight gain in male and female WT and miR-146a-/- mice on HFD for 11 weeks. (G-I) C57BL6/J (blue) and miR-146a-/- (green) mice purchased from Jackson Laboratories were placed on HFD and the following were measured: (G) percent weight gain, (H) body weight gain (in grams), and (I) food consumption measured both day and night at 0, 3 and 18 weeks HFD in metabolic chambers. p-values were calculated using two-tailed Student’s t-test. *p<0.05; **p<0.01; ***p<0.001; ****p<0.0001.
(TIF)

S2 Fig miR-146a and BAT weight and gene expression. (A-C) qRT-PCR expression data from BAT samples of young, untreated WT (blue) or miR-146a-/- (green) mice relative to L32 expression in (A) BAT activation genes, (B) Lipogenesis genes, and (C) inflammatory immune genes. (D) Weight (g) of BAT samples from WT or miR-146a-/- mice. (E) qRT-PCR expression of miR-146a relative to 5s in WT (blue) or miR-146a-/- (green) BAT samples. (F) qRT-PCR expression data from BAT samples of WT (blue) or miR-146a-/- (green) mice following HFD, relative to L32 expression for a number of BAT and inflammatory genes. Data are shown as mean ± SEM (n = 5). p-value was calculated using two-tailed Student’s t-test. *p<0.05; **p<0.01.
(TIF)

S3 Fig miR-146a protects against high blood glucose levels during diet-induced obesity but does not alter pancreatic architecture. (A) WT and miR-146a-/- mice on NCD or HFD were injected with glucose at 0 minutes and blood glucose levels were measured over time for 120 minutes. (B) Blood glucose of 6-hour fasted WT and miR-146a-/- mice on NCD or HFD. (C) H&E staining of representative sections of pancreas at week 14 of diet treatment. Data are shown as mean±SEM or as individual mice; p-value was calculated using two-tailed Student’s t-test. *p<0.05; **p<0.01.
(TIF)

S4 Fig Increased weight gain by miR-146a-/- mice during DIO is not dependent upon miR-155. (A) Percent weight gain over time of diet in WT, miR-155-/-, miR-146a-/-, and DKO mice on HFD. (B) Body weight (in grams) of WT, miR-155-/-, miR-146a-/-, and DKO mice over time of diet. (C) Blood glucose levels of WT, miR-155-/-, miR-146a-/-, and DKO mice following a six-hour fast, at 15 weeks HFD. (D) Weight of reproductive, visceral fat pads harvested from WT, miR-155-/-, miR-146a-/-, and DKO mice following HFD. (E) TD-NMR body composition measurement showing percent body fat of WT, miR-155-/-, miR-146a-/- mice at week 14 HFD. (F) Percent lean mass of total body weight in WT, miR-155-/-, miR-146a-/-, and DKO mice at week 14 HFD. Data are shown as mean±SEM (n = 5); p-value was calculated using two-tailed Student’s t-test. *p<0.05; **p<0.01; ***p<0.001; ****p<0.0001.
(TIF)

S5 Fig GSEA of RNA-seq data from miR-146a-/- and WT mouse ATMs on NCD or HFD. (A) Percentages of live, singlet CD45^+^ cells positive for CD11b and F4/80 markers, collected from the SVF of VAT in WT and miR-146a-/- mice fed NCD or HFD. (B) Total number of live, singlet, CD45^+^ cells positive for CD11b and F4/80 markers, collected from the SVF of VAT in WT and miR-146a-/- mice fed NCD or HFD. (C) Percentage of live, singlet CD45+ cells and percentage of CD45+ B (B220+) and T (CD3e+) cells, from the SVF of VAT in WT and miR-146a-/- mice fed HFD. (D) Gene Sets significantly upregulated in miR-146a-/- HFD mice compared with WT, according to GSEA. (E) Gene sets significantly upregulated in miR-146a-/- NCD mice compared with WT, according to GSEA. NES = normalized enrichment score; FDR = false discovery rate, where FDR<0.25 is statistically significant. For a and b, p-values were calculated using two-tailed Student’s t-test. *p<0.05; ns = not significant.
(TIF)

S1 Table Materials table listing all materials used in this publication. (PDF)

S2 Table Underlying numeric data. (XLSX)
