# Supplementary figures and images for: Anti-inflammatory microRNA-146a protects mice from diet-induced metabolic disease
Source: PLoS Genet. 2019 Feb 15;15(2):e1007970. doi: 10.1371/journal.pgen.1007970 (PMC6395003; doi:10.1371/journal.pgen.1007970)

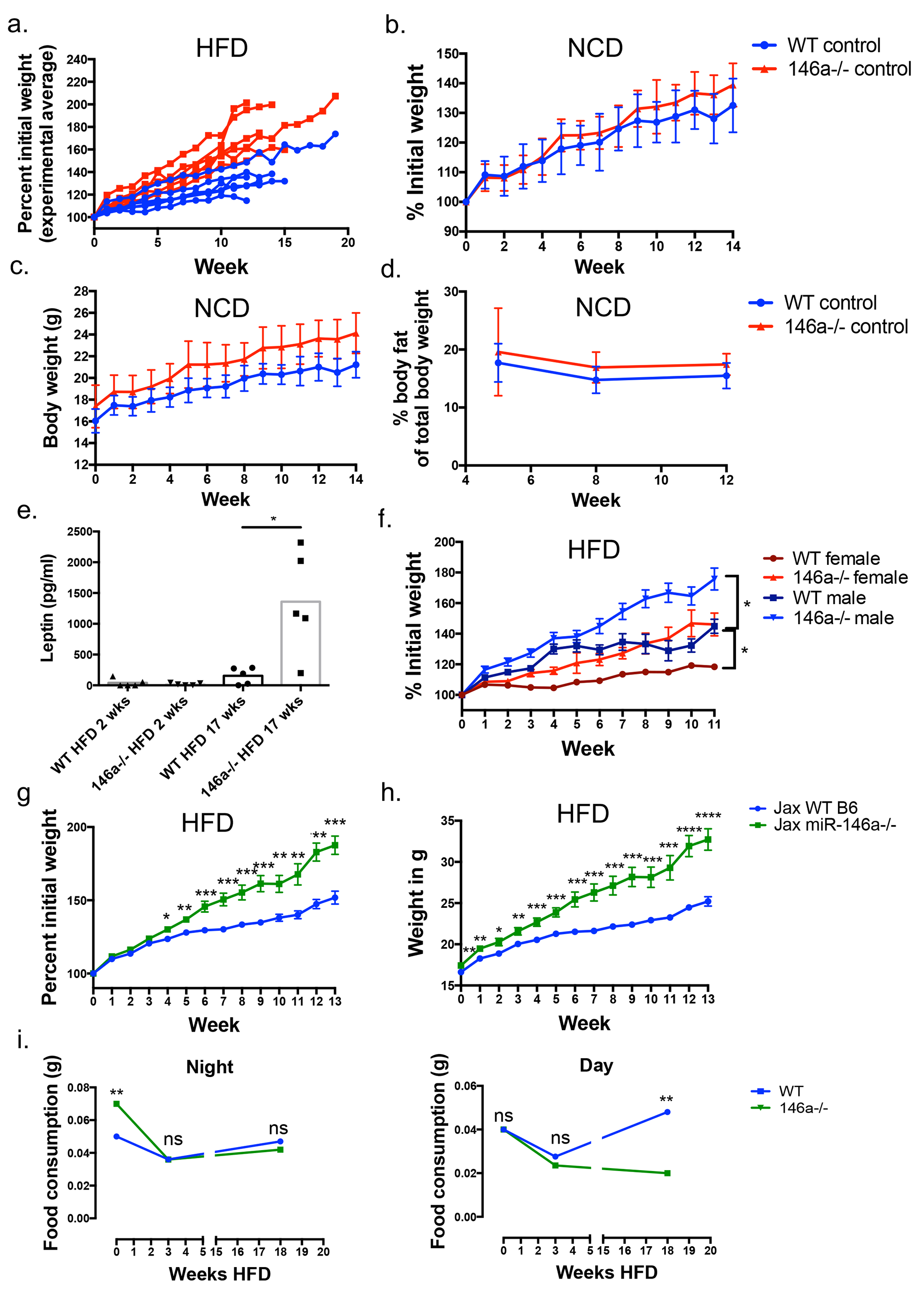

Supplement: S1 Fig — (A) Each line shows average percent weight gain over time during an individual experimental repeat of young female WT (blue) and miR-146a-/- (red) mice on HFD. (B) Percent weight gain over time of WT and miR-146a-/- mice on NCD. (C) Body weight gain (in grams) over time of WT and miR-146a-/- mice on NCD. (D) TD-NMR body composition measurements showing percent body fat of WT and miR-146a-/- mice at 5, 8, and 12 weeks of NCD. (E) Leptin protein levels from serum of 6-hour fasted WT and miR-146a-/- mice at 2 and 17 weeks HFD. (F) Comparison of percent weight gain in male and female WT and miR-146a-/- mice on HFD for 11 weeks. (G-I) C57BL6/J (blue) and miR-146a-/- (green) mice purchased from Jackson Laboratories were placed on HFD and the following were measured: (G) percent weight gain, (H) body weight gain (in grams), and (I) food consumption measured both day and night at 0, 3 and 18 weeks HFD in metabolic chambers. p-values were calculated using two-tailed Student’s t-test. *p<0.05; **p<0.01; ***p<0.001; ****p<0.0001. (TIF) [file pgen.1007970.s001.tif]

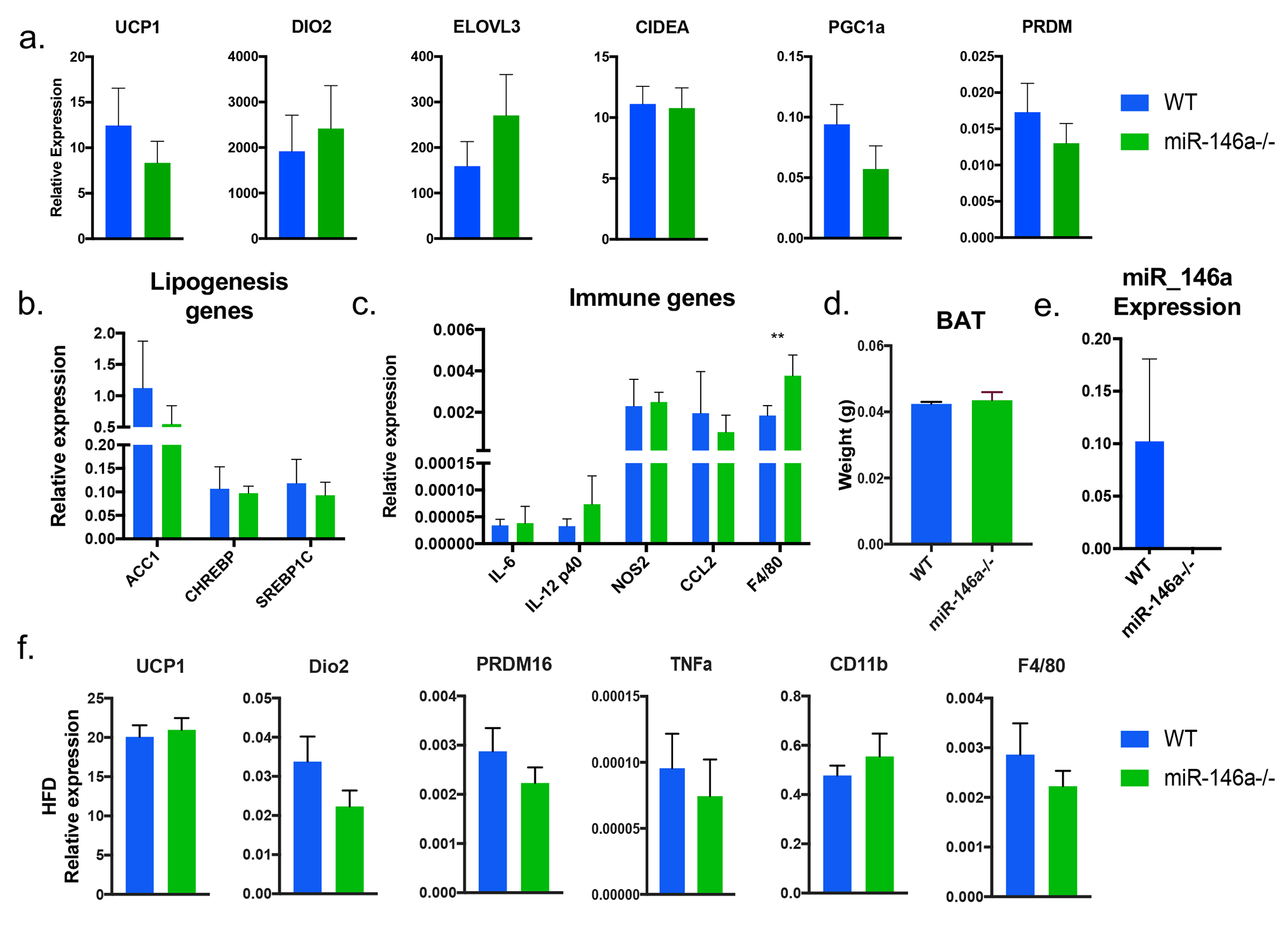

Supplement: S2 Fig — (A-C) qRT-PCR expression data from BAT samples of young, untreated WT (blue) or miR-146a-/- (green) mice relative to L32 expression in (A) BAT activation genes, (B) Lipogenesis genes, and (C) inflammatory immune genes. (D) Weight (g) of BAT samples from WT or miR-146a-/- mice. (E) qRT-PCR expression of miR-146a relative to 5s in WT (blue) or miR-146a-/- (green) BAT samples. (F) qRT-PCR expression data from BAT samples of WT (blue) or miR-146a-/- (green) mice following HFD, relative to L32 expression for a number of BAT and inflammatory genes. Data are shown as mean ± SEM (n = 5). p-value was calculated using two-tailed Student’s t-test. *p<0.05; **p<0.01. (TIF) [file pgen.1007970.s002.tif]

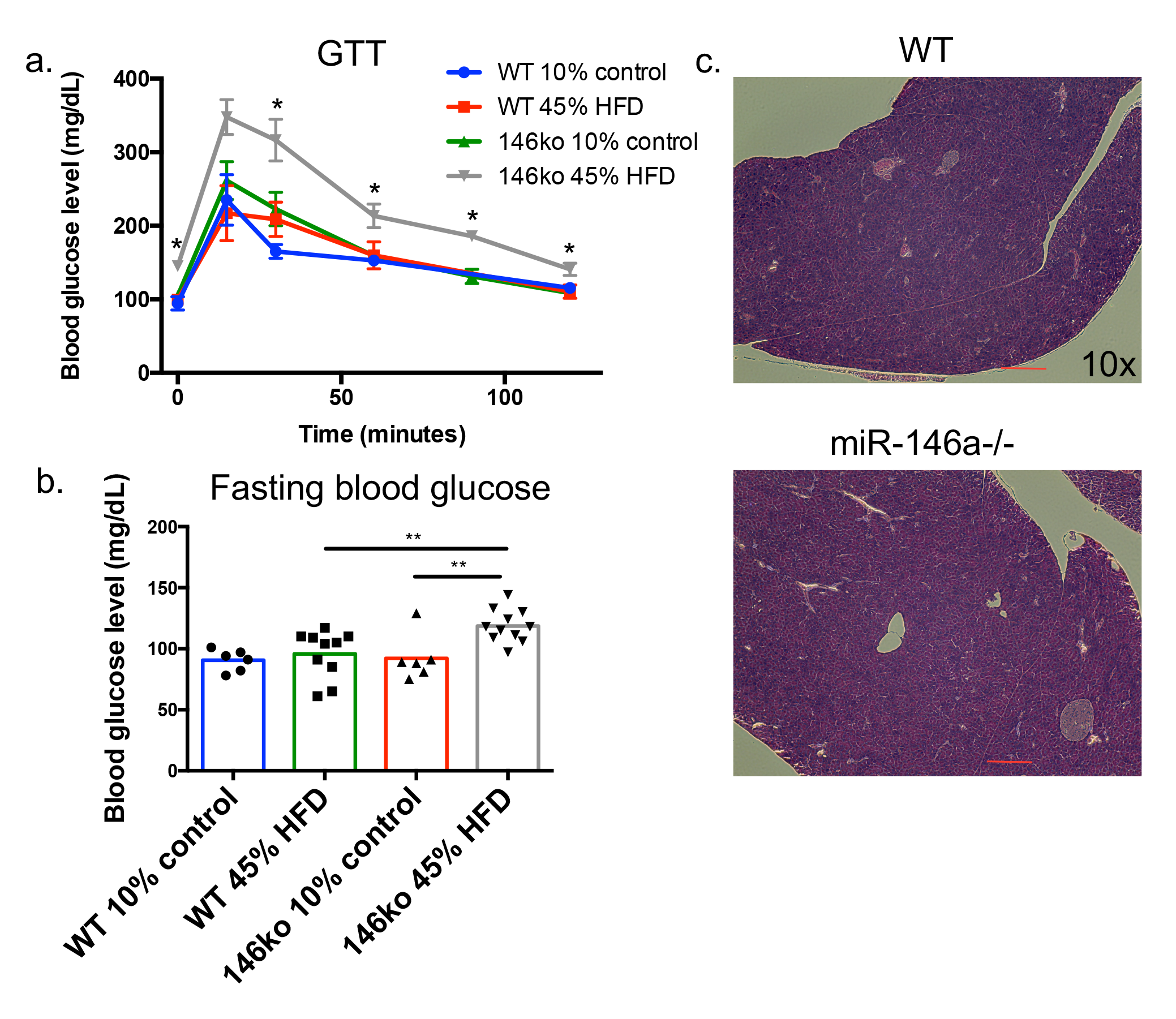

Supplement: S3 Fig — (A) WT and miR-146a-/- mice on NCD or HFD were injected with glucose at 0 minutes and blood glucose levels were measured over time for 120 minutes. (B) Blood glucose of 6-hour fasted WT and miR-146a-/- mice on NCD or HFD. (C) H&E staining of representative sections of pancreas at week 14 of diet treatment. Data are shown as mean±SEM or as individual mice; p-value was calculated using two-tailed Student’s t-test. *p<0.05; **p<0.01. (TIF) [file pgen.1007970.s003.tif]

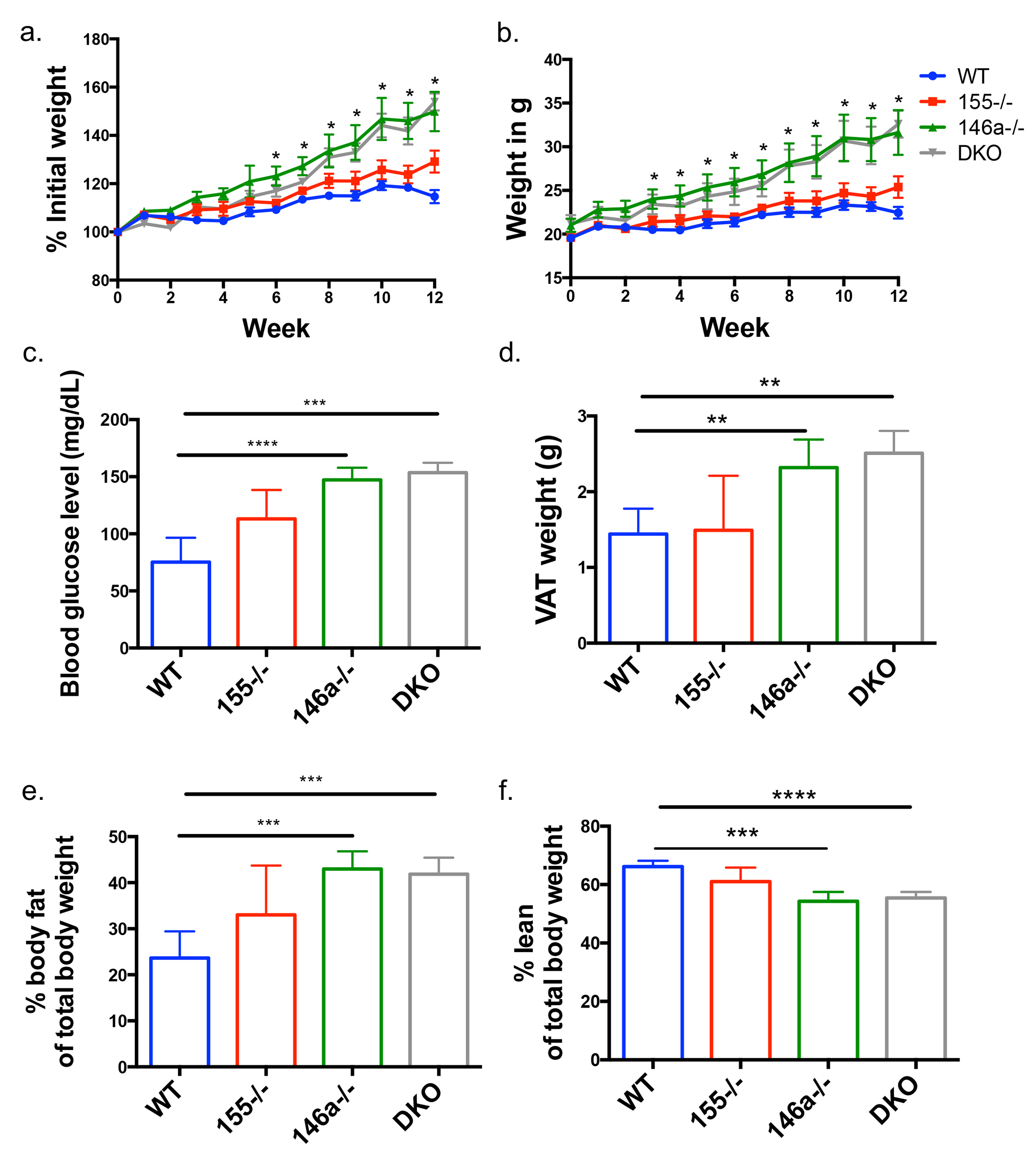

Supplement: S4 Fig — (A) Percent weight gain over time of diet in WT, miR-155-/-, miR-146a-/-, and DKO mice on HFD. (B) Body weight (in grams) of WT, miR-155-/-, miR-146a-/-, and DKO mice over time of diet. (C) Blood glucose levels of WT, miR-155-/-, miR-146a-/-, and DKO mice following a six-hour fast, at 15 weeks HFD. (D) Weight of reproductive, visceral fat pads harvested from WT, miR-155-/-, miR-146a-/-, and DKO mice following HFD. (E) TD-NMR body composition measurement showing percent body fat of WT, miR-155-/-, miR-146a-/- mice at week 14 HFD. (F) Percent lean mass of total body weight in WT, miR-155-/-, miR-146a-/-, and DKO mice at week 14 HFD. Data are shown as mean±SEM (n = 5); p-value was calculated using two-tailed Student’s t-test. *p<0.05; **p<0.01; ***p<0.001; ****p<0.0001. (TIF) [file pgen.1007970.s004.tif]

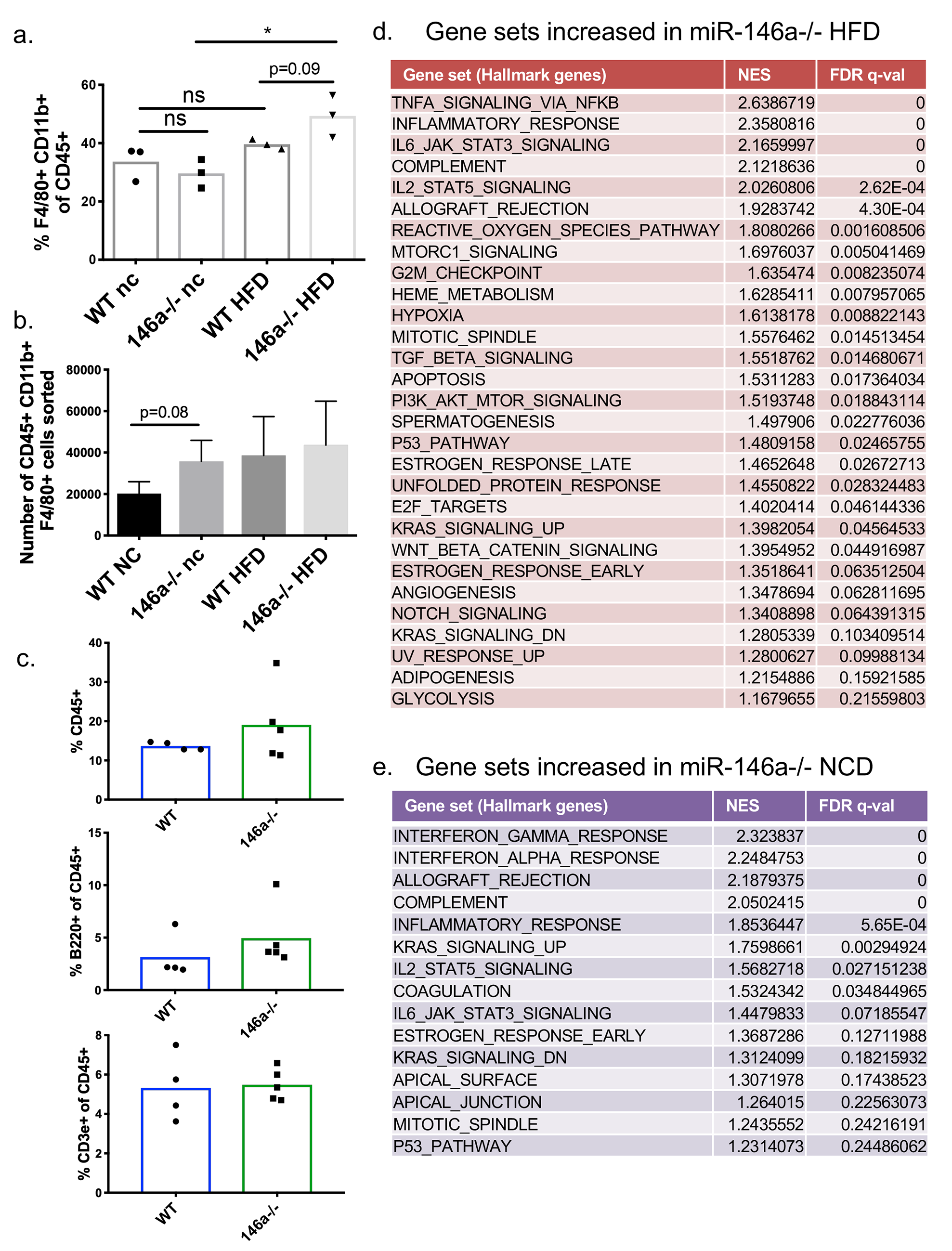

Supplement: S5 Fig — (A) Percentages of live, singlet CD45+ cells positive for CD11b and F4/80 markers, collected from the SVF of VAT in WT and miR-146a-/- mice fed NCD or HFD. (B) Total number of live, singlet, CD45+ cells positive for CD11b and F4/80 markers, collected from the SVF of VAT in WT and miR-146a-/- mice fed NCD or HFD. (C) Percentage of live, singlet CD45+ cells and percentage of CD45+ B (B220+) and T (CD3e+) cells, from the SVF of VAT in WT and miR-146a-/- mice fed HFD. (D) Gene Sets significantly upregulated in miR-146a-/- HFD mice compared with WT, according to GSEA. (E) Gene sets significantly upregulated in miR-146a-/- NCD mice compared with WT, according to GSEA. NES = normalized enrichment score; FDR = false discovery rate, where FDR<0.25 is statistically significant. For a and b, p-values were calculated using two-tailed Student’s t-test. *p<0.05; ns = not significant. (TIF) [file pgen.1007970.s005.tif]
