# Supplementary material for: Anti-inflammatory microRNA-146a protects mice from diet-induced metabolic disease
Source: PLoS Genet. 2019 Feb 15;15(2):e1007970. doi: 10.1371/journal.pgen.1007970 (PMC6395003; doi:10.1371/journal.pgen.1007970)
Supplement: S1 Table — (PDF) [file pgen.1007970.s006.pdf]

S1 Table: Materials Table

REAGENT or RESOURCE	SOURCE	IDENTIFIER
Antibodies		
phosphorylated IKBα	Cell Signaling	Cat#9246S; RRID:
IKBα	Cell Signaling	Cat#9242S; RRID:
Traf6	Abcam	Cat#ab33915; RRID: AB_778572
beta actin	Abcam	Cat#ab8226; RRID:AB_306371
GAPDH	Santa Cruz	Cat#sc-25778 RRID:AB_1016766 8
PerCP/Cy5.5 anti-mouse CD45 Antibody	BioLegend	Cat#103131 RRID:AB_893344
APC CD11b	BioLegend	Cat#101212 RRID:AB_312795
PE anti-mouse F4/80 [BM8] Antibody	BioLegend	Cat#123110 RRID:AB_893486
Chemicals, Peptides, and Recombinant Proteins		
Glucose	Sigma	G8270
Recombinant Mouse M-CSF (carrier-free)	Biolegend	Cat#576404
GoTaq Master Mix	Promega	Cat#A6002
Lipopolysaccharides from Escherichia coli 026:B6	Sigma Chemical	Cat#L5543-2ML
Rapamycin	LC Laboratories	Cat#R-5000
CD40-Traf6 Signaling Inhibitor	Millipore Sigma	Cat#5.30352.0001
Captisol	Ligand	Cat#RC-0C7-100
PEG400	Sigma-Aldrich	Cat#202398-250G
Critical Commercial Assays		

Seahorse XF-96 Mito Stress Test	Agilent	https://www.agilent.com/en/products/cell-analysis/seahorse-analyzers/seahorse-xfe96-analyzer
Ultra Sensitive Mouse Insulin ELISA	Crystal Chem	Cat#90080
miRNeasy Mini Kit	Qiagen	Cat#217004
qScript Flex cDNA Synthesis Kit	Quanta Biosciences, VWR	Cat#101414-110
MAGPIX mouse metabolic hormone panel	Merck Millipore	Cat#MMHMAG-44K
Deposited Data		
RNA-Sequencing Data in ATMs	NCBI GEO Repository	Refer to materials and methods in paper
Experimental Models: Cell Lines		
RAW 264.7 Cell line	ATCC	
Experimental Models: Organisms/Strains		
M. musculus: Strain C57BL/6 miR-146a-/-	This Paper	N/A
M. musculus: Strain C57BL/6 WT	This Paper	N/A
M. musculus: Strain C57BL/6 miR-146a-/-	The Jackson Laboratory	Stock no.: 016239
M. musculus: Strain C57BL/6 WT	The Jackson Laboratory	Stock no.: 000664
Oligonucleotides		
Traf6 Crispr guide #1: GGAGATCCAGGGCTACGATG	This paper	N/A
Traf6 Crispr guide #2: GATGGAACTGAGACATCTCG	This paper	N/A
hsa-miR-146a-5p LNA miRNA PCR Assay (Primer set, UniRT)	Qiagen	Cat#339306
Cxcl10<br>F: GAAATCATCCCTGCGAGCCTATCC<br>R: GCAATTAGGACTAGCCATCCACTGGG	IDT	N/A
Tnfa<br>F: CCCTCACACTCAGATCATCTTCT<br>R: GCTACGACGTGGGCTACAG	IDT	N/A
MHCII (*H2-Ab1* exon 1/2)<br>F: CTGCCATTACCTGTGCCTTAG<br>R: GTAGCACTCGCCCATGAACT	IDT	N/A
F4/80 (*Adgre1*)<br>F: TGGGAAAGACTGGATTCTGGG<br>R: GGAGCCATTCAAGACAAAGCC	IDT	N/A

CD11b (*Itgam*)<br>F: CCTTCATCAACACAACCAGAGTGG<br>R: CGAGGTGCTCCTAAAACCAAGC	IDT	N/A
Cd36<br>F: TCCTCTGACATTTGCAGGTCTATC<br>R: AAAGGCATTGGCTGGAAGAA	IDT	N/A
Fabp4<br>F: AAGAAGTGGGAGTGGGCTTTG<br>R: CTGTCGTCTGCGGTGATTTC	IDT	N/A
Il12p40<br>F: AGATGACATCACCTGGACCT<br>R: GCCATGAGCACGTGAACCGT	IDT	N/A
Ccl2<br>F: TAAAAACCTGGATCGGAACCAAA<br>R: GCATTAGCTTCAGATTTACGGGT	IDT	N/A
Traf6<br>F: AAGCCTGCATCATCAAATCC<br>R: CTGGCACTTCTGGAAAGGAC	IDT	N/A
Nos2<br>F: CAGCTGGGCTGTACAAACCTT<br>R: CATTGGAAGTGAAGCGGTTCG	IDT	N/A
IL-6<br>F: TAGTCCTTCCTACCCCAATTTCC<br>R: TTGGTCCTTAGCCACTCCTTC	IDT	N/A
CD45 (PTPRC)<br>F: GGGTCCACCTACATAAATGCCAG<br>R: GTTCCTGTTTCCTTCTTCACATCG	IDT	N/A
Leptin<br>F: GAGACCCCTGTGTCGGTTC<br>R: CTGCGTGTGTGAAATGTCATTG	IDT	N/A
Ucp1<br>F: CGTACCAAGCTGTGCGATGT<br>R: GAGTCGCAGAAAAGAAGCCAC	IDT	N/A
Dio2<br>F: CTGCGCTGTGTCTGGAAC<br>R: GGAGCATCTTCACCCAGTTT	IDT	N/A
Elovl3<br>F: TCCGCGTTCTCATGTAGGTCT<br>R: GGACCTGATGCAACCCTATGA	IDT	N/A
Cidea<br>F: ATCACAACTGGCCTGGTTACG<br>R: TACTACCCGGTGTCCATTTCT	IDT	N/A

Pgc1a<br>F: CCCTGCCATTGTTAAGACC<br>R: TGCTGCTGTTCCTGTTTTC	IDT	N/A
Prdm16<br>F: CAGCACGGTGAAGCCATTC<br>R: GCGTGCATCCGCTTGTG	IDT	N/A
Acc1<br>F: TGTACAAGCAGTGTGGGCTGGCT<br>R: CCACATGGCCTGGCTTGGAGGG	IDT	N/A
Chrebp<br>F: GGCCTGGCTGGAACAGTA<br>R: CGAAGGGAATTCAGGACAGT	IDT	N/A
Srebp1c<br>F: GGCCCGGGAAGTCACTGT<br>R: GGAGCCATGGATTGCACATT	IDT	N/A
Software and Algorithms		
Prism 7	GraphPad Software	https://www.graphpad.com/company/
Illumina HiSeq 50 cycle single-read sequencing version 4	University of Utah High Throughput Genomics Core Facility	N/A
Other		
10% kcal control mouse feed	Research Diets	D12450Bi
45% kcal diet, irradiated	Research Diets	D12451i
